# Symptom progression in neuromyelitis optica spectrum disorder from ataxia through syncope to neuropathic pain: A case report

**DOI:** 10.1097/MD.0000000000038966

**Published:** 2024-07-19

**Authors:** Ziyi Zhao, Chunhua Pan, Junting Chen, Rui Wu, Zucai Xu, Hao Huang

**Affiliations:** a Department of Neurology, Affiliated Hospital of Zunyi Medical University, Zunyi, China.

**Keywords:** acute brainstem syndrome, ataxia, neuromyelitis optica spectrum disorder, neuropathic pain, syncope, Wallenberg syndrome

## Abstract

**Rationale:**

Neuromyelitis optica spectrum disorder (NMOSD) involves autoimmune and inflammatory responses in the central nervous system, primarily affecting the optic nerves and spinal cord. Atypical presentations such as ataxia and syncope complicate the diagnosis, and lesions in the medulla are easily mistaken for cerebral infarction. This case report emphasizes the need to recognize such manifestations to avoid misdiagnosis and ensure timely treatment.

**Patient concerns:**

This case report presents an NMOSD female patient who experienced ataxia, syncope, and neuropathic pain during her illness.

**Diagnosis:**

NMOSD.

**Interventions:**

The patient managed her blood sugar with insulin, controlled neuropathic pain with pregabalin, and underwent 5 plasma exchanges.

**Outcomes:**

Significant improvement was noted 1 week post-plasma exchange, with complete resolution of neuropathic pain and no symptom recurrence reported at 6-month follow-up.

**Lessons:**

Atypical manifestations of NMOSD, such as ataxia, syncope, and trigeminal neuralgia, increase diagnostic difficulty. Recognizing these symptoms is crucial to avoid misdiagnosis and ensure timely and appropriate treatment for patients.

## 1. Introduction

Neuromyelitis optica spectrum disorders (NMOSD) are central nervous system disorders characterized by autoimmune-mediated inflammation and demyelination, predominantly affecting the optic nerves and spinal cord. NMOSD manifests through 6 principal symptoms: acute optic neuritis, acute myelitis, area postrema syndrome (characterized by unexplained episodes of hiccups and/or nausea and vomiting lasting at least 48 hours or with magnetic resonance imaging (MRI) evidence of dorsal brainstem lesions), acute brainstem syndrome, symptomatic narcolepsy or acute diencephalic clinical syndrome with NMOSD-specific diencephalic MRI lesions, and symptomatic cerebral syndrome with NMOSD-specific brain lesions.

In clinical practice, NMOSD cases presenting with severe optic neuritis and acute myelitis as initial symptoms are relatively straightforward to diagnose. However, acute brainstem syndrome, despite being 1 of the 6 core symptoms, is less frequently observed compared to optic neuritis and acute myelitis. The patient described in this article initially presented with ataxia as the primary symptom. As the disease progressed, the appearance of ataxia, syncope, and neuropathic pain increased the diagnostic complexity, leading to delays in treatment and management.

## 2. Case report

A 64-year-old female patient was admitted to our hospital after experiencing sudden dizziness and an unstable gait 10 days prior. The episodes of transient dizziness lasted several hours and were accompanied by intermittent nausea and vomiting. She exhibited a noticeable rightward lean while walking. Despite undergoing treatment at a local hospital, there was no improvement; her symptoms of gait instability even worsened, leading her to seek care at our institution.

**Figure 1.**
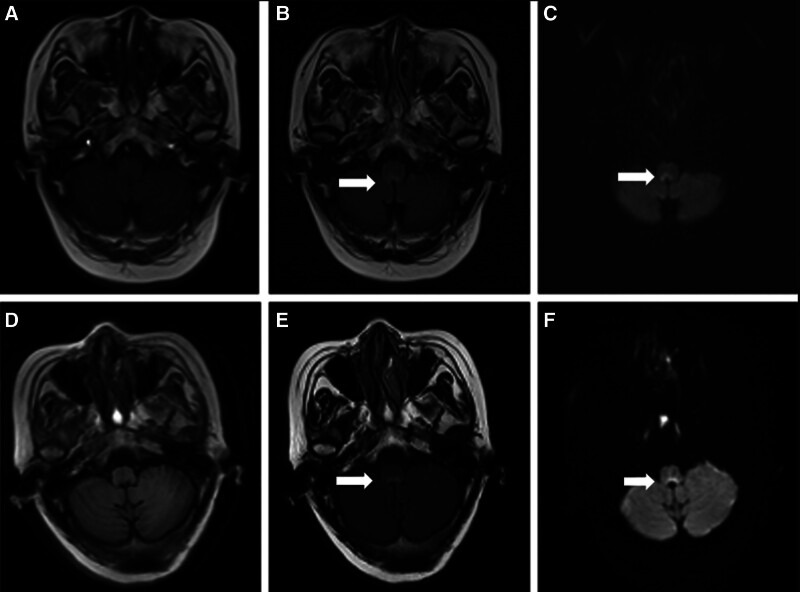
(A–C) A small area of prolonged T2 signal intensity is observed in the dorsal medulla oblongata. Scattered areas of prolonged T2 signal are visible in both frontal lobes. There is enlargement of the ventricles, with widening of the sulci, fissures, and cisterns, while the midline structures remain aligned. (D–F) A similar small area of prolonged T2 signal is noted in the dorsal region of the medulla oblongata, with scattered prolonged T2 signal areas also present in both frontal lobes. The ventricles are enlarged, and there is widening of the sulci, fissures, and cisterns, but the midline structures are again aligned. The lesion in the dorsal medulla oblongata appears reduced in size compared to the initial imaging.

The patient has a 6-year history of diabetes, which has been managed with insulin therapy to control blood glucose levels. One week prior to onset, she had an upper respiratory infection characterized by coughing and a runny nose. Neurological examination revealed horizontal nystagmus, reduced pharyngeal reflexes, mild dysarthria, right-sided ataxia, and right-sided muscle strength of grade 4. The right-sided finger-to-nose and heel-to-shin tests were imprecise, and the Romberg test (eyes open and closed) was positive, making it impossible for her to walk in a straight line. Blood glucose level was 7.22 mmol/L. Routine blood tests, coagulation profile, electrolytes, antinuclear antibodies, anti-SSA/SSB antibodies, and anticardiolipin antibodies showed no abnormalities. Cranial MRI indicated a lesion on the dorsal side of the medulla, suggestive of a minor infarction (Fig. 1A–C). Cranial CT and cervical MRI scans were normal. Head and neck magnetic resonance angiography (MRA) showed no intracranial arterial abnormalities, but revealed irregular thickness in the right vertebral artery, with visible irregularities in the V1 and V4 segments. Given the sudden onset of symptoms, the patient’s age, and her diabetic status, a provisional diagnosis of a cerebral infarction, specifically dorsolateral medullary syndrome, was made. Treatment included 100 mg of aspirin daily, 20 mg of atorvastatin calcium nightly, and continued insulin for glycemic management. Following this treatment regimen, the patient reported gradual alleviation of her dizziness and was able to ambulate independently with crutches on an intermittent basis.

During her hospital stay, the patient experienced several episodes of transient syncope in the early night hours, between 0:00 and 2:00 am. Each episode began with brief nausea and discomfort, subsequently leading to a loss of consciousness and collapse. There were no convulsions or frothing at the mouth, and each episode resolved spontaneously within approximately 30 seconds.

Following thorough discussions between the healthcare team and the patient along with her family, it was advised to conduct cerebrospinal fluid screening and tests for central nervous system demyelinating diseases, especially given the patient’s history of exposure to cold and flu-like symptoms before the onset of her current condition, raising concerns for potential infectious or immune-mediated CNS disorders. However, the patient and her family declined these recommendations. Based on the patient’s existing tests and assessments, her episodes of nocturnal transient syncope were preliminarily attributed to dorsolateral medullary syndrome. After a week of hospitalization and treatment, the patient was discharged, reporting an overall improvement in her condition.

One month after discharge, the patient suddenly began experiencing unprovoked bilateral facial and head pain, resembling needle pricks, each lasting about 1 minute. Despite receiving carbamazepine and other treatments for trigeminal neuralgia, there was no improvement in the pain’s intensity or frequency, prompting her return to our hospital for further treatment. Neurological examination revealed a leftward deviation of the tongue upon protrusion, diminished pharyngeal reflexes, normal limb muscle tone, muscle strength graded at 5, hyperactive limb tendon reflexes, right-sided ataxia, and no pathological signs. Cranial MRI revealed degeneration and softening of the dorsal medulla; ischemic lesions in the white matter of both frontal lobes; and a reduction in the extent of dorsal medullary lesions (Fig. 1D–F). MRI scans of the cervical–thoracic spine and trigeminal nerves were normal. Neurophysiological examinations showed abnormal sympathetic skin responses (SSR) in both hands.

Integrating the patient’s comprehensive medical history, including the temporal and spatial aspects of her medication use, alongside the presentation of neuropathic pain, the potential for a demyelinating lesion within the central nervous system was considered. After discussions with the patient and her family, consent was obtained to conduct cerebrospinal fluid analysis and tests for CNS demyelinating diseases, encompassing both serum and cerebrospinal fluid assessments. The cerebrospinal fluid analysis revealed a pressure of 80 cm H_2_O and a protein level of 534 mg/L, with no abnormalities detected in routine tests or bacterial smears. The serum test for CNS demyelinating disease indicated a positive AQP4-IgG at a titer of 1:100, leading to a definitive diagnosis of NMOSD. The patient was treated with pregabalin for pain management and underwent plasma exchange. Following 5 plasma exchange sessions, there was a significant improvement in her cephalo-facial pain symptoms. A telephone follow-up 6 months later confirmed the complete resolution of her head and facial pain, with the patient regaining full independence in walking.

## 3. Conclusions

NMOSD is a rare immune-mediated, central nervous system-targeted autoimmune inflammatory and demyelinating disease that primarily involves the optic nerve and spinal cord, also known as Devic disease.^[1]^ Its prevalence varies across different study populations, ranging from 0.52 to 4.4 per 100,000 individuals.^[2]^ The prevalence of NMOSD appears to be higher in East Asians (Japanese, Chinese, and Koreans) compared to whites and other Asian ethnic groups (approximately 3.5/100,000).^[3]^

NMOSD is an autoimmune disease of the central nervous system with a predominantly humoral immune profile, a spectrum of autoimmune CNS inflammatory demyelinating disorders closely associated with antibodies to aquaporin-4 (AQP4). AQP4 is extensively distributed in the central nervous system, particularly within the brain, spinal cord, and optic nerves,^[4]^ and is predominantly situated in regions adjacent to the cerebrospinal fluid. This includes the astrocytic foot processes at the blood–brain barrier, as well as in other locations such as the kidneys’ collecting ducts, the stomach’s mural cells, airways, secretory glands, and skeletal muscles. The brain, in particular, is more vulnerable to antibody-mediated immune attacks due to the absence of complement inhibitory mechanisms found in other body organs.^[5]^ In the brain, AQP4 is located in areas in contact with cerebrospinal fluid and is specifically localized to the peduncles of astrocytes at the blood–brain barrier.^[4]^ Anti-AQP4 antibodies, primarily of the IgG1 subtype, specifically target the AQP4 antigen. This interaction prompts AQP4-expressing astrocytes to release interleukin 6, which compromises the integrity of the blood–brain barrier. This leads to complement-mediated and cell-mediated damage to the astrocytes, undermining their support and protection of peripheral cells like oligodendrocytes and neurons. The ensuing inflammatory response results in significant astrocyte damage and death, along with secondary damage to oligodendrocytes and neurons, characterized by demyelination.^[2]^

NMOSD is characterized by 6 primary clinical manifestations: optic neuritis, acute myelitis, area postrema syndrome, acute brainstem syndrome, symptomatic narcolepsy or acute diencephalic clinical syndrome, and symptomatic cerebral syndrome.^[6]^ Diagnosing NMOSD is relatively straightforward when the initial symptoms are optic neuritis and myelitis. Data from a large international NMOSD cohort study reveal that during the disease’s progression, myelitis occurs in 84% of patients, optic neuritis in 63%, area postrema syndrome in 15%, brainstem syndrome in 17%, diencephalic syndrome in 3%, and cerebral syndrome in 14%.^[7]^ Notably, the incidence of initial acute brainstem syndrome presentations is significantly higher in Asian populations compared to European American populations.^[8]^

In this case, the patient’s illness onset followed a cold, underscoring the significant role infections play in NMOSD pathophysiology. Viral infections play a role in increasing the permeability of the blood–brain barrier, allowing antibodies to penetrate it more readily, and viruses can also activate the immune system through molecular mimicry, promoting an autoimmune response.^[9]^ Over the disease’s trajectory, patients frequently encounter painful symptoms, with pain prevalence exceeding 80% among those with NMOSD. The spectrum of pain experienced includes neuropathic, nociceptive, and mixed types, manifesting during acute flare-ups or evolving into chronic conditions throughout the disease.^[10]^ In the brainstem, the dorsal and extreme posterior regions of the medulla oblongata have the highest distribution of AQP4. Neuropathic pain is more common in NMOSD patients with medullary lesions than in those without such lesions.^[11]^

When a patient with NMOSD presents with initial symptoms in the dorsolateral medulla oblongata, it can readily be misdiagnosed as a cerebrovascular event, specifically dorsolateral medulla oblongata syndrome. And when the patient’s symptoms of nausea and eructation are not obvious, it is easy to consider the patient’s medullary cerebral infarction as the cause and ignore the patient’s further treatment. When the patient simply has facial neuropathic pain, the typical symptoms of NMOSD are not obvious. And medullary dorsolateral syndrome is also a typical cause of post-stroke central pain, which may sometimes be characterized by trigeminal neuralgia,^[12]^ leading to more neglect of the patient’s primary etiology. In this case, symptoms such as ataxia, syncope, and facial neuralgia masked the patient’s ultimate diagnosis, leading to delayed treatment. Additionally, because a cerebrospinal fluid (CSF) examination is invasive, the patient and her family initially refused to undergo CSF testing, which limited a comprehensive understanding of her condition and more accurate diagnosis. This underscores the importance of patient compliance in case management. It must be acknowledged that the limitations of this study, including the lack of extensive biomarker analysis and long-term follow-up, mean that the long-term effects of treatment interventions and the potential risks of relapse may not have been fully assessed.

In summary, in this case report, we discuss an NMOSD case with atypical clinical manifestations, particularly highlighting the patient’s unconventional symptoms, which added complexity to the diagnosis. While NMOSD primarily affects the optic nerves and spinal cord of the central nervous system, this case demonstrates the diversity of disease symptoms, necessitating consideration of such diagnostic variations. For this patient, clinical symptoms improved after treatment with aspirin, but caution is needed due to the occurrence of syncope at night. Identifying these atypical manifestations is crucial to avoiding misdiagnosis and ensuring that patients receive timely and appropriate treatment.

## Author contributions

**Supervision:** Hao Huang, Zucai Xu.

**Validation:** Hao Huang.

**Writing – original draft:** Ziyi Zhao, Rui Wu.

**Writing – review & editing:** Ziyi Zhao.

**Data curation:** Chunhua Pan, Junting Chen.

**Formal analysis:** Chunhua Pan, Junting Chen.
